# p38β and Cancer: The Beginning of the Road

**DOI:** 10.3390/ijms21207524

**Published:** 2020-10-12

**Authors:** Olga Roche, Diego M. Fernández-Aroca, Elena Arconada-Luque, Natalia García-Flores, Liliana F. Mellor, María José Ruiz-Hidalgo, Ricardo Sánchez-Prieto

**Affiliations:** 1 Laboratorio de Oncología, Unidad de Medicina Molecular, Centro Regional de Investigaciones Biomédicas, Unidad Asociada de Biomedicina UCLM, Unidad Asociada al CSIC, Universidad de Castilla-La Mancha, 02008 Albacete, Spain; Olga.roche@uclm.es (O.R.); DiegoManuel.Fernandez@alu.uclm.es (D.M.F.-A.); elena.arconada@uclm.es (E.A.-L.); natalia.garcia16@alu.uclm.es (N.G.-F.); liliana.mellor@gmail.com (L.F.M.); maria.rhidalgo@uclm.es (M.J.R.-H.); 2 Departamento de Ciencias Médicas, Facultad de Medicina de Albacete, Universidad de Castilla-La Mancha, 02008 Albacete, Spain; 3 Departamento de Química Inorgánica, Orgánica y Bioquímica, Área de Bioquímica y Biología Molecular, Facultad de Medicina de Albacete, Universidad de Castilla-La Mancha, 02008 Albacete, Spain; 4 Departamento de Biología del Cáncer, Instituto de Investigaciones Biomédicas Alberto Sols (CSIC-UAM), Unidad Asociada de Biomedicina UCLM, Unidad Asociada al CSIC, Consejo Superior de Investigaciones Cientificas, 28029 Madrid, Spain

**Keywords:** p38MAPK, MAPK11, p38β, cancer

## Abstract

The p38 mitogen-activated protein kinase (MAPK) signaling pathway is implicated in cancer biology and has been widely studied over the past two decades as a potential therapeutic target. Most of the biological and pathological implications of p38MAPK signaling are often associated with p38α (MAPK14). Recently, several members of the p38 family, including p38γ and p38δ, have been shown to play a crucial role in several pathologies including cancer. However, the specific role of p38β (MAPK11) in cancer is still elusive, and further investigation is needed. Here, we summarize what is currently known about the role of p38β in different types of tumors and its putative implication in cancer therapy. All evidence suggests that p38β might be a key player in cancer development, and could be an important therapeutic target in several pathologies, including cancer.

## 1. Introduction

Mitogen-activated protein kinases (MAPKs) are an evolutionarily conserved family of enzymes that link extracellular signals to the intracellular machinery in order to control a plethora of cellular processes including proliferation, cell survival, differentiation and apoptosis, among others. In fact, its deregulation is associated with many human diseases including inflammation, neurodegenerative disorders and cancer [1].

In mammals, four conventional MAPK subfamilies have been identified: extracellular signal-regulated protein kinases 1/2 (ERK1/2), c-Jun N-terminal kinases 1-3 (JNK1/2/3), p38MAPKs (α, β, γ and δ), and the most recently discovered and least characterized ERK5 [1]. Each MAPK has its own activators, inhibitory phosphatases, substrates and scaffold proteins that allow the correct function of the different MAPK signaling pathways [2,3]. The diversity and specificity of MAPKs in cellular responses are achieved with a linear architecture, consisting of a module of three protein kinases: a MAPK kinase kinase (MAP3K or MKKKs) at the top, which phosphorylates a MAPK kinase (MAP2K, MKKs or MEKs) on specific serine (S) and threonine (T) residues. Eventually, there is a dual phosphorylation of the T and tyrosine (Y) residues of the conserved T-X-Y motif, located in a loop close to the active site of the terminal MAPK [4]. 

In addition, there is the group of atypical MAPKs, including ERK3/4, ERK7, ERK8 and Nemo-like kinase (NLK) [5,6,7,8], whose regulation and activation is not related to the module of the three kinases described for the conventional ones. 

## 2. The p38MAPK Family

In mammalian cells, the p38MAPK family includes four members: p38α (MAPK14), p38β (MAPK11), p38γ (MAPK12) and p38δ (MAPK13), which have a high degree of sequence homology at the amino acid level (>60%) [9]. p38MAPKs differ in their expression patterns and substrate specificities, suggesting diverse functions. The p38MAPKs are S/T proline-directed kinases with an activation motif, T-G-Y, in which the substrate specificity is not only determined by the targeted amino acids, but also by specific docking domains present on the substrate and by a specific substrate binding motif in the MAPK (for a recent review of the p38MAPK-mediated signaling see [10]). The primary MAP2Ks for the p38MAPKs modules are MKK3 and MKK6 [11], although initially it was also considered the activation through MKK4 [12]. Activation of MAP2Ks occurs by phosphorylation of two conserved S and T residues on their activation loop by a broad range of MAPK3s. The MAP3Ks of this pathway include ASK1 (apoptosis signal-regulating kinase 1), DLK1 (dual-leucine-zipper bearing kinase 1), TAK1 (transforming growth factor β-activated kinase 1), TAO (thousand and one amino acid) 1 and 2, TPL2 (tumor progression loci 2), MLK3 (mixed-lineage kinase 3), MEKK (3 and 4), and ZAK1 (leucine zipper and sterile-α motif kinase 1) [13]. However, it has also been reported activation/inactivation of this signaling pathway by non-canonical mechanisms as in the case of T-cell receptor [14] or GRK2 [15]. 

The p38MAPK family can be divided into two subsets: on the one hand, p38α and p38β, and on the other hand p38δ and p38γ. This classification is based on their homology and their susceptibility to be inhibited by pyridinyl imidazoles (SB203580 and SB202190 compounds) at low concentrations. p38α and p38β have a higher homology between them (75%) and both can be inhibited by pyridinyl imidazoles, whereas p38δ and p38γ are 61% and 62% identical to p38α, respectively, and are not susceptible to be inhibited by SB203580 and SB202190 [16,17]. The number of specific inhibitors for p38MAPK is rapidly growing, allowing for a better understanding of the biological role of each p38 family member [18]. Important substrates in the p38MAPK signaling pathway include downstream kinases such as MK2/3, PRAK MSK1 or MNK1/2, as well as various transcription factors including ATF1/2/6, c-Myc, c-Fos, GAT4A, MEF2A/C, SRF, STAT1, p53 and CHOP among others [19]. This diversity of factors associated with the p38MAPK signaling pathway gives a glimpse of the plethora of biological processes implicated in this pathway.

## 3. p38β

p38β (also known as Stress Activating Protein Kinase 2 (SAPK2), Stress Activating Protein Kinase 2b (SAPK2b), MAPK11 or P38β2) was described in 1996 by Jiang and coworkers [20], and is encoded by the *MAPK11* gene that maps to chr22:50,263,713-50,270,380 in the human genome (UCSC genome browser/GRCh38/hg38), and comprises 12 exons (NCBI reference sequence NM_0022751.7). The protein is 364 amino acids long, and has a kinase domain (amino acids 24-308) that includes a T-G-Y (amino acids 180-182) dual phosphorylation motif, which is required for its kinase activity [16,21]. The 3D structure of p38β resembles that of a typical kinase with a smaller β-sheet N-terminal domain and a larger C-terminal domain. The ATP-binding site is located between the two domains, which are linked by a single polypeptide chain (residues T107-G110). This structure is also very similar to that of p38α, although they differ in the relative orientation of the N- and C-terminal domains [22]. This orientation causes a reduction in the size of the ATP-binding pocket of p38β compared to p38α. The difference in size between the two pockets could play a role in their different substrate specificity [20], and could be exploited in order to design selective compounds able to inhibit each p38 protein independently [22]. 

p38β is ubiquitously expressed, but at lower levels than p38α. p38β is expressed in the human brain, heart, placenta, lung, liver, skeletal muscle, kidney, spleen, testis, ovary, prostate, thymus and pancreas [20,21]. Moreover, p38β is abundant in endothelial cells, but undetectable in other lineages as macrophages or monocytes [23]. Similar to p38α, p38β is activated by pro-inflammatory cytokines and environmental stress, such as IL-1β, TNF, sorbitol, arsenite, anisomycin, high osmolarity, H_2_O_2_ and UV light [16,24]. The MAP2K that activates p38β is MKK6, whereas p38α is activated by MKK3 and MKK6 [20,25,26]. A unique characteristic of p38β is the ability to modulate its basal activity by autophosphorylation events. p38β is capable of self-activation by cis autophosphorylation of the residue T180 located in the activation loop. This activation occurs spontaneously in vitro, but can be regulated in mammalian cell cultures [27]. Moreover, p38β also autophosphorylates in trans residues T241 and S261 in vivo. Indeed, phosphorylation of S261 reduces the activity of T180-phosphorylated p38β, whereas, T241 phosphorylation reduces its phosphorylation in trans, although these two phosphorylation events do not seem to affect the activity of dually phosphorylated (T180/Y182) p38β [28]. 

The functions of p38β are mostly redundant with those of p38α. For instance, it has been shown that p38β cannot perform specific functions of p38α during development [29]. In fact, p38α knockout mice are lethal due to placental defects [30], while p38β knockout mice are fertile and viable [31].

The substrates attributed to p38β are mainly based on the use of SB compounds, which inhibit both p38α and p38β, not allowing to determine if they are bona fide substrates of p38β (p38MAPK substrates are reviewed in [10]). However, there are p38β targets that have been confirmed with other approaches. Among the several p38β substrates, there are protein kinases, transcription factors, and transcriptional regulators. Regarding protein kinases, the MAPK-activated protein kinases MAPKAPKs are a group of proteins downstream of MAPKs. A subgroup of MAPKAPKs is composed of MK2, MK3 (also known as 3pK), and MK5 (also designated as p38-regulated/activated protein kinase (PRAK)). These three kinases regulate key cellular processes such as gene expression at the transcriptional and post-transcriptional level, control cytoskeletal architecture and cell cycle progression, and play an important role in pathological processes such as inflammation and cancer (reviewed in [32]). p38α and p38β inhibit mitotic entry through MK2/3 phosphorylation in vivo [33] and MK2/MK3 activation is blocked by the inhibitor SB203580 in vitro [34]. MK5 is activated by p38α and p38β in vitro and in vivo [35], regulating the shuttling of this protein from the nucleus to the cytoplasm [36]. Another substrate of p38β is Protein kinase C Є (PKCЄ), a serine/threonine kinase involved in the regulation of cytokinesis in mitotic cells. This protein is primed to bind 14-3-3 by a series of phosphorylation events initiated by p38MAPK (in S350), GSK3 (in S346) and PKC itself (in S368). In vitro studies have shown that p38α and p38β phosphorylate S350 creating a GSK3 recognition site for the phosphorylation of S346, and that chemical inhibition by SB203580 prevents S346 phosphorylation in cells stimulated by UV-C [37]. 

Other studies have shown that p38β is also associated with several transcription factors. For example, MEFs (Myocite Enhancer Factors) are a family of transcription factors composed of MEF2A-D that regulates cell differentiation, proliferation, apoptosis, migration, and metabolism [38]. MEF2A and MEF2C are phosphorylated by p38α and p38β in vitro through a MAP kinase docking domain that is specific to these MAPKs, and activates their transcriptional activity in vivo [39]. Moreover, SB202190 inhibits the transcriptional activity of MEF2C induced by LPS or MKK6 in monocytic cells [40]. Another transcription factor targeted by p38β is NFATc4 (Nuclear Factor of activated T cells 4). NFATc4 belongs to the NFAT family of transcription factors, and is involved in cardiac development, mitochondrial function, and in activation of adipocyte specific genes during differentiation [41,42]. NFATC4 is phosphorylated by p38α, β, γ and δ in the presence of an activated MKK6 mutant (MKK6-GLu) in vitro and in vivo, p38α phosphorylates NFATc4 at S168 and S170 in the NFAT homology domain regulating the subcellular distribution of the transcription factor, promoting cytoplasmic localization of the NFATc4, and blocking adipocyte formation under differentiation conditions [43]. Moreover, phosphorylation of S168 and S170 of endogenous NFATc4 by p38MAPK is sensitive to SB203580 [44]. 

AP-1, a dimeric complex that is composed of members of the JUN, FOS, ATF or MAF protein families, regulates a wide range of cellular processes including cell proliferation, death, survival and differentiation, and has also been shown to be a downstream target of p38β [45]. C-FOS and ATF2 were also shown to be phosphorylated in vitro and in vivo by the four p38MAPKs, increasing its transcriptional activity [16,21,46,47]. Furthermore, it was reported that histone deacetylase 3 interacts specifically with p38β in LPS-stimulated cells, diminishing its phosphorylation, and leading to a repression of ATF-2 transcriptional activity as in the case of TNF gene expression [48]. Another transcription factor targeted by p38β is MafA, a member of the MAF family of basic leucine zipper proteins, that act as an important regulator of development and differentiation in many organs/tissues, and is a key player in Insulin regulation (for a review see [49]). MafA is also phosphorylated by the four p38MAPK isoforms in vitro and in vivo, and this phosphorylation might control MafA function, as it was shown previously in lens differentiation in primary cultures of chicken neuroretinal cells [50].

Other substrates of p38β with different functions that are shared with p38α, have been also reported. This group of miscellaneous substrates includes the BAF 60 protein BAF60c [51,52], E47 [53], P18 (Hamlet) [54,55], Cyclin D3 [56], the variant of the histone H2A, H2AX [57], KH-type splicing regulatory protein (KSRP) [58], and the membrane associated metalloprotease TACE [59]. However, there are two proteins, Glycogen Synthase (GS) and Raptor that seem to be specific substrates of p38β, and are not phosphorylated by any of the other p38MAPK proteins. p38β binds specifically to GS in skeletal muscle, brain and liver, and its efficient phosphorylation allows GSK3 to phosphorylate other residues of GS, causing partial inhibition of its activity [60]. In the case of Raptor, a regulatory-associated protein of mTOR, activated p38β by arsenite interacts with Raptor resulting in the phosphorylation of Raptor on S863 and S771, enhancing mTORC1 activity [61]. Therefore, the search for new specific substrates based on genetic evidence rather than on SB compounds, is a key step in the further understanding of biological functions mediated by p38β. 

## 4. p38β and Cancer

Although p38β has been related to several pathological conditions like Huntington disease [62] and cardiac hypertrophy [63,64], this review will be focused on the role of p38β in cancer. 

Since the mid-90s, when the p38MAPK signaling pathway was initially related to the cellular response to DNA damage agents including antitumor treatments [65], up to recent evidence indicating its use as a potential therapeutic target [13], the role of the p38MAPK signaling pathway in cancer has been deeply studied. However, most of the work has been focused on p38α, which has been repeatedly shown to play an important role in cancer biology. Consistent data from experimental models in different pathological conditions [66,67], have allowed us to consider p38α as a biomarker [24,68,69], and also as a putative target for cancer therapy (for a review see [70]). Conversely, much less is known about the role of the other p38 proteins (p38β/γ/δ) in cancer, although recent studies have shown an important role of p38γ/δ in cancer [71,72], however, further studies are needed to elucidate the definitive role of these two p38 proteins in cancer pathology (for a review see [73]).

Little is known about the role of p38β in cancer, although this protein has been associated with key molecules in this disease. For example, p38β has been proposed as a key target of the proto-oncogene Pokemon, a transcription factor known to be implicated in tumorigenesis and metastasis in hepatic cells [74]. Also, it has been reported that p38β could be a critical step in tumor formation through regulation of lipocalin 2 (LCN2) expression, a direct target of Plakophilin 3 (PKP3). In this sense, it has been shown that in different types of tumors, high LCN2 expression correlates with increased invasion, tumor formation and metastasis (for a review see [75]). Interestingly, in the absence of PKP3, p38β is able to control the expression of LCN2, indicating a potential role of p38β in tumor formation [76]. p38β has also been associated with integrin-αv, known to maintain cellular proliferation in keratinocytes by controlling c-Myc translation through FAK, p38β and p90RSK1. Chemical inhibition of p38β or genetic interference of *MAPK11* in keratinocytes promotes a marked decrease in c-Myc levels [77]. It was proposed that p38β could play a key role in biological processes for tumor progression and angiogenesis. For instance, TGF-β1 was shown to induce endothelial cell apoptosis by changing VEGF signaling from p38β, with survival function, to p38α with a pro-apoptotic function [78], in agreement with previous observations in cardiomyocytes [79]. Other studies have shown a direct connection of p38β with VEGF in a murine retinal model [80], further highlighting the importance of p38β in neovascularization and hypoxia-induced cell proliferation. Altogether, these studies suggest that p38β could be a potential target for an anti-angiogenic approach.

Also, p38β has been related to other aspects associated to cancer disease, with important implications in the patient’s quality of life. For example, p38β has been related to cachexia through the control exerted onto the autophagic protein ULK1 in both in vitro and in vivo muscle wasting models [81]. Indeed, it is known that p38β functions upstream of FoxO–BNIP3 signaling axis to mediate an energy stress response [82], supporting the role of this MAPK in energy sensing. Another interesting aspect of p38β is its relationship with cancer-associated pain. In an experimental model of rats, pain associated to intra-tibial injection of mammary gland carcinoma cells, showed a marked reduction by intrathecal administration of a p38β antisense oligonucleotide [83]. Furthermore, the reduction of cancer-associated pain by music therapy was also attributed to low expression of p38β and p38α [84].

In addition to the connection with key proteins and biological processes in cancer, there are several examples showing the implication of p38β in different types of tumors. In pancreatic cancer, Singh and coworkers reported that p38β could be a potential biomarker [85]. Furthermore, the authors showed that peptide inhibitors for p38β are able to induce toxicity in pancreatic cell lines such as PANC-1, suggesting a potential therapeutic implication [85]. In hepatocellular carcinoma, recent data showed that p38β is a target of miR-516a-5p, which is controlled by a novel circular RNA, circ-0001955, that increases the expression of p38β, facilitating hepatocellular tumorigenesis [86]. In bladder cancer, p38β has been reported to be a critical player in cell motility through the signaling axis ILK-p38β-Hsp27 [87]. In prostate cancer, p38β has been related to metastases through the control exerted on the Wnt inhibitor Dickkopf-1, indicating the possibility of being considered as a therapeutic target [88]. Another study considered that the p38α/β inhibitor SB202190 could be used as a putative therapy in this type of tumor, in which STK11 could be a critical biomarker for this p38-based therapy, but no genetic evidence supported a critical role for p38β [89]. Therefore, further investigation is necessary to clarify the role of this particular MAPK in the biology and therapy of prostate cancer. In endometrial cancer, p38β has been shown to mediate the proliferation of tumor cells by inhibiting apoptosis. In this case, the anti-apoptotic ability of p38β seems to be controlled by the long non-coding RNA 1220, that controls p38β expression [90]. Interestingly, in lung cancer it has been recently reported that p38α, but not the rest of p38MAPK members, could be a potential biomarker of chemotherapy response [68]. However, overexpression of p38β was shown to be related to a specific subset of lung cancer in non-smokers in China [91]. Other reports indicate that a single-nucleotide polymorphism in p38β (rs2076139) is a potential biomarker associated with progression-free survival in metastatic non-small-cell lung cancer patients receiving platinum-based chemotherapy [92]. In addition, in lung cancer of non or light smokers it was shown that p38β and p38α, could be predictors of the expression levels of the DNA excision repair protein ERCC1, a key protein in DNA damage reparation with implications for the response to platinum compounds [93]. Indeed, chemical inhibition of p38α/β decreased viability of lung cancer cell lines, but genetic interference showed that most of this effect relies on p38β [93]. However, in terms of response to cisplatin, the effect of p38β was not applicable to all the experimental models [93], suggesting a more prominent role for p38α. Nonetheless, further studies are required to fully elucidate the role of p38β in lung cancer and its therapy. In breast cancer, the only connection with p38β has been related to bone metastases, through the up-regulation of the expression and secretion of monocyte chemotactic protein-1, which activates osteoclast differentiation and activity. Interestingly, the authors show how targeting p38β in breast cancer cells could be a novel approach to treat bone destruction associated with bone metastasis [94]. In silico evidence connected triple-negative breast cancer with epirubicin response and p38β overexpression, among other MAPKs, but no experimental data have been provided so far [95]. In Head and Neck Squamous Cell Carcinoma (HNSCC) patients, high expression levels of all p38MAPK isoforms, including p38β, have been detected in sera. Interestingly, these levels are downregulated after therapy, except for p38δ, suggesting that all p38MAPKs could be potential biomarkers in this disease. In addition, the authors indicated a potential role of p38δ as a putative target for HNSCC therapy that cannot be extrapolated to p38β [96]. 

p38β has also been related to leukemic pathology, for example, in acute myeloid leukemia (AML) and in Sézary syndrome. In AML, an aggressive hematologic malignancy, the overexpression of the SET oncoprotein, able to inhibit the protein phosphatase PP2A, is a key event that correlates with poor prognosis [97]. In this regard, p38β has been associated with the inhibitory effect of SET onto PP2A by two different mechanisms: first, by promoting SET cytoplasmic translocation through CK2 phosphorylation, and second, by direct binding to and stabilization of the SET protein [98]. Therefore, and considering the anticancer activity of several PP2A-activating drugs [99], p38β could be a potential novel target in AML, especially in those cases with SET over-expression. In the Sézary syndrome, a leukemic variant of cutaneous T-cell lymphomas, it has been reported that the overexpression of p38β could be a potential driver gene or a novel biomarker [100]. Indeed, in Sézary syndrome-derived cell lines, inhibition of PKCβ and GSK3 with the small molecules Enzastaurin and AR-A014418 promote a marked decrease in p38β expression without changing p38α levels [100]. Also, SB203580 and SB202190 promote cell death in those cell lines as well as in primary samples from Sézary syndrome patients. However, the genetic interference of p38β does not show any effect in cell viability [100], suggesting that further studies are necessary to fully evaluate the potential therapeutic implications of p38β in Sézary syndrome. 

Nevertheless, in other types of tumors, p38β appears not to have any implication or, if so, a marginal role. For example, in melanoma, preliminary evidence in cell lines discard this MAPK, but not p38α, as a key player in this pathology [101]. Another example could be colorectal cancer, in which the 1628A>G (rs2235356) genetic variation in the p38β promoter region may contribute to the susceptibility to colorectal cancer in a Chinese population [102]. However, recent reports discard specifically this result in a Swedish population [103], suggesting that maybe p38β is not a universal biomarker for colorectal cancer. Indeed, other screening study discards p38β and indicates that p38α could be considered as a potential diagnostic marker and a putative therapeutic target for colorectal cancer [104]. In fact, this last observation is in agreement with previous reports using patient-derived xenografts [105]. Altogether, all this evidence suggests a marginal role for p38β in colorectal cancer.

Finally, regarding the implications of p38β in cancer biology, it is important to mention that Stress Activated Protein Kinases signaling pathways, JNK and p38MAPK, have been shown to play a dual role in cancer, both as an oncogene and as a tumor suppressor gene (for a review see [106,107]). This dual role seems to be dependent on several factors, including the experimental model and the stage of cell transformation, among others. Colorectal cancer is a paradigmatic example of this duality, showing that p38α could behave as an oncogene or a tumor suppressor depending on the stage of the carcinogenesis process [66]. It is likely that similar to p38α, p38β may have a dual role in cancer, also playing a tumor suppressor role. For instance, p38 MAPK signaling has been proposed to act as a tumor suppressor gene by controlling oncogenic properties of key molecules such as Ras (reviewed in [108]), Wip1 [109], EGFR [110]), and urokinase plasminogen activator [111] among others. However, most of these studies address the function of p38α specifically, with no reference to p38β, or if so, discarding its implication in the tumor suppressor activity. Future work investigating the potential tumor suppressing activity of p38β is needed to fully understand if p38β can potentially act both as a proto-oncogene and as a tumor suppressor gene in cancer pathology. 

From the therapeutic point of view, the implication of p38MAPK signaling pathway in the mechanism of action of several anti-cancer drugs has been widely studied, but most of these studies have focused on the role of p38α [112]. In fact, p38α has been connected to DNA damage-response [113] through its relation with key proteins in DNA damage such as ATM or p53 [114]. p38α has been proposed as a master regulator of the apoptotic effects triggered by genotoxic drugs [115], and also as a central part of the cellular response to ionizing radiation [116]. However, no data involving specifically p38β has been published. There are only few examples suggesting a role for p38β in response to cancer therapy. For instance, in leukemia-derived cell lines, both p38α and p38β have been linked to interferon-α, leading to an inhibition of the cellular growth [117]. Moreover, p38β has been proposed as a key molecule in the stimulation of cell death triggered by the p38α/β inhibitor SB202190, UV, and FasL indicating a role in cytotoxicity [118]. Regarding other commonly used cancer treatments, such as chemo/radiotherapy or immunotherapy, there are no studies addressing the implications of p38β in response to these treatments, except for the one mentioned above in lung cancer [93], and for thymoquinone, a natural compound, in which its antitumor effect has been related to down regulation of p38β [119]. The relevance “per se“ of p38β in cancer treatments, as a putative target, has been demonstrated in the previously mentioned experimental model of pancreatic cancer by using specific p38β inhibitory peptides [85] but, unfortunately, no other examples of specific targeted therapy based on p38β have been reported so far.

## 5. Future Directions

**Figure 1:**
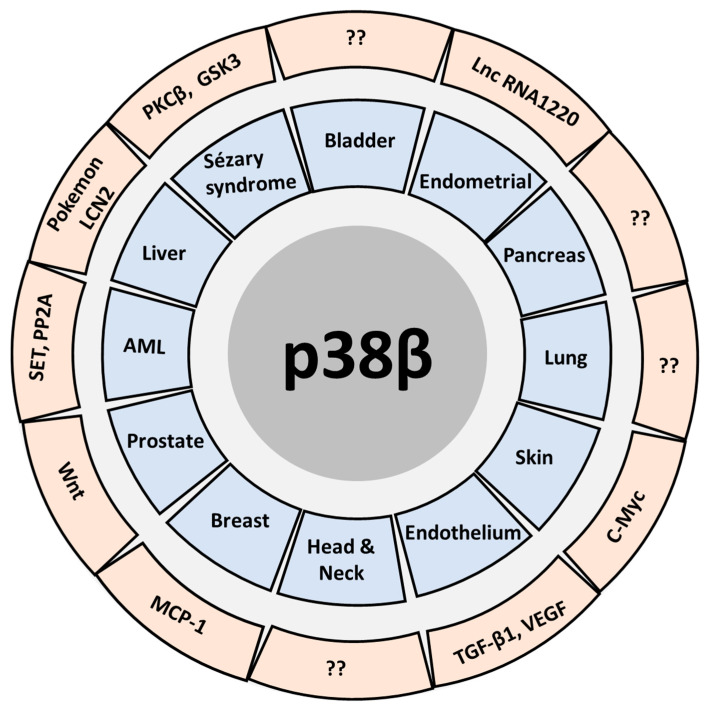
Schematic representation of the involvement of p38β in different types of tumors (blue) and the related molecules (red).

Although p38β is the least studied member of the p38MAPK family, possibly due to its functional redundancy with p38α, recent evidence shows that it may play a differential role with biological and pathological implications, as in the case of cancer. The lack of specific inhibitors for this MAPK has greatly complicated its study, since it involves the use of genetic approaches almost on a mandatory basis. Undoubtedly, the development of specific inhibitors for p38β could accelerate the research of this MAPK. However, there are still aspects to be investigated in the coming years such as the role of p38β in transcriptional regulation, its specific substrates, its involvement in the process of cell transformation and cancer (Figure 1), its implication in the cellular response to chemo and radiotherapy treatments, or even its use as a putative therapeutic target. Our knowledge of p38β is increasing every day, similarly to other members of the p38 family, allowing us to unravel the complexity of p38MAPK signaling, and to further elucidate the specific roles of each p38 family member. However, as most of the current research is focused on p38α, further studies on other p38 proteins, including p38β are needed to fully understand the importance of the p38MAPK signaling in human pathology.
